# The next frontier of immunotherapy: clinical trial prospects for lung cancer vaccines

**DOI:** 10.1097/JS9.0000000000004054

**Published:** 2026-03-04

**Authors:** Qing-Dong Zhu, Chang Song, Chun-Yan Zhao, Chang-Yue Jiang, Hang-Biao Qiang, Xiao-Mei Yang, Yi-Bo Lu, Zhou-Hua Xie

**Affiliations:** a Department of Tuberculosis, The Fourth People’s Hospital of Nanning, Nanning, Guangxi, China; b Clinical Medical School, Guangxi Medical University, Nanning, Guangxi, China; c Department of Pharmacy, The Fourth People’s Hospital of Nanning, Nanning, Guangxi, China; d Department of Radiology, The Fourth People’s Hospital of Nanning, Nanning, Guangxi, China

Therapeutic cancer vaccines, an active immunotherapy strategy, activate the patient’s immune system to specifically recognize and eliminate tumor cells, offering the potential for durable antitumor responses. As such, they have become a key area of investigation in lung cancer treatment^[1]^. Since William Coley’s pioneering work in the early 20th century, cancer vaccines have undergone more than a century of development, emerging as one of the most promising directions in oncology research and clinical translation^[2]^. This article systematically presents a comprehensive analysis of global clinical trial data on lung cancer vaccines, highlighting trends in development, trial design characteristics, and clinical outcomes, thereby providing a scientific foundation for the future optimization of lung cancer immunotherapy.

Our study conducted a comprehensive analysis of clinical trial progress related to lung cancer vaccines based on the INFORMA Pharma Database (https://pharma.id.informa.com/). We developed a core search strategy centered on the terms “lung cancer” and “cancer vaccine.” Two independent reviewers screened the records and extracted relevant data, and discrepancies were resolved by a third reviewer through discussion. A total of 455 clinical trials meeting the inclusion criteria were identified and analyzed. Key dimensions of analysis included temporal trends, geographic distribution, funding sources, vaccine types, molecular targets, and mechanisms, as well as trial phases and status. To ensure the reproducibility of the study, we have detailed the complete search query used in the INFORMA Pharma database in the supplementary file, including all relevant filtering conditions and key search details (Supplemental Digital Content 1, available at: http://links.lww.com/JS9/G955). In this study, all aspects of the research, including the design, data analysis, and derivation of conclusions, were exclusively and independently executed by the researchers without the use of any artificial intelligence (AI) tools^[3]^.

The evolution of lung cancer vaccine development from 1991 to 2025 reveals distinct phases. The early years (1991–2000) were characterized by a predominance of Phase I trials, marking preliminary explorations in this field. Between 2005 and 2015, the steady rise in both Phase I and II trials reflected an expansion of research efforts. More recently (2021–2025), there has been a resurgence in Phase III trials, notably with five trials registered in 2024, indicating that several vaccine candidates have advanced to critical validation stages (Fig. 1A). Additionally, traditional trial designs often struggle to adequately evaluate the delayed immune effects and long-term benefits associated with cancer vaccines. Regulatory hurdles further complicate the pathway to approval (Fig. 1B). As shown in Table 1, the number of completed Phase I trials significantly exceeds those of later phases, indicating relatively active and successful early-stage investigations.

**Figure 1.**
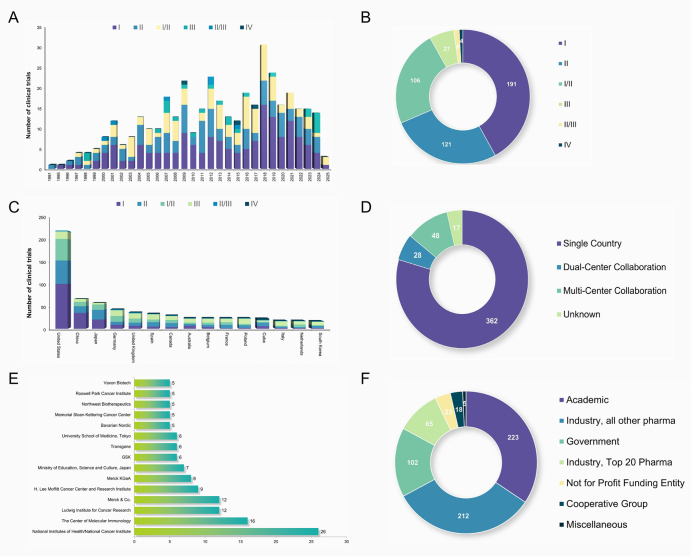
Overview of clinical trials for lung cancer vaccines. (A) Annual distribution of clinical trials for lung cancer vaccines from 1991 to 2025. (B) Overall distribution of different trial types. (C) Distribution of the top 15 countries conducting clinical trials for lung cancer vaccines. (D) Collaborative landscape of clinical trials for lung cancer vaccines. (E) Distribution of the top 15 sponsors for clinical trials of lung cancer vaccines. (F) Types of funding organizations supporting clinical trials for lung cancer vaccines.

**Table 1:** Characteristics of lung cancer vaccine trials.

Characteristics	I	II	I/II	III	II/III	IV
Completed	113	65	68	10	1	4
Terminated	35	39	24	9	5	0
Open	22	13	5	6	0	0
Planned	12	2	3	2	0	0
Closed	9	1	4	0	0	0
Temporarily closed	0	1	2	0	0	0

The United States continues to lead global research and development efforts, supported by a mature biomedical infrastructure and strong venture capital investment. China and several European countries also demonstrate notable contributions. Notably, Cuba has registered 23 trials, standing out among developing nations, likely due to its unique policy environment and national prioritization of vaccine research (Fig. 1C). In terms of collaboration patterns, single-center studies dominate the landscape, comprising 79.56% of all trials. Dual-center (6.15%) and multicenter (10.55%) collaborations remain limited, reflecting persistent regional tendencies and barriers to international cooperation (Fig. 1D). Strategic policy reforms aimed at fostering cross-border collaboration may help address these limitations and enhance global scientific exchange. Regarding funding sources, academic institutions lead the field, sponsoring 201 trials. Pharmaceutical companies outside the top 20 in global rankings account for 173 trials, significantly outnumbering the top 20 companies, which are responsible for just 36 trials. Government agencies support 60 trials, while non-profit organizations (16 trials) and collaborative groups (13 trials) also play meaningful roles, highlighting a diversified funding landscape shaped by academia, industry, and public-sector involvement (Fig. 1E, F). In the early period (1995–2009), government-led programs demonstrated sustained investment in foundational areas such as antigen screening, yet trials in Phase III or beyond constituted less than 4% of the total, highlighting their exploratory, basic research orientation (Supplemental Digital Content Tables S1–S2, available at: http://links.lww.com/JS9/G954). In contrast, industry-funded initiatives since 2015 have shown an explosive concentration on non-small cell lung cancer (NSCLC). This focus complements the government-led efforts, forming a cohesive research and development chain. Collectively, these patterns underscore the necessity of multi-stakeholder collaboration to overcome the persistent challenges in clinical efficacy (Supplemental Digital Content Tables S3–S4, available at: http://links.lww.com/JS9/G954).

Technologically, epitope-based peptide vaccines and cell-based vaccines constitute the two major pillars of lung cancer vaccine development. Together, these categories represent 67% of the top 15 most actively developed vaccine candidates (Fig. 2A). Target selection in clinical trials exhibits significant clustering effects (Fig. 2B). Programmed cell death 1 (PD-1) and EGFR are the most frequently targeted, appearing in 23% and 18% of studies, respectively. This dominance reflects two key factors: first, the clinical success of PD-1/PD-L1 immune checkpoint inhibitors, which has validated the therapeutic value of this pathway, and second, the extensive data available on EGFR, a known oncogenic driver in lung cancer, derived from the long-standing clinical use of small-molecule inhibitors such as gefitinib. This combination of “mechanistic validation” and “data continuity” makes these targets preferred candidates for vaccine development. In biomarker selection, CD8A (a marker for cytotoxic T cells) and CD4 (a marker for helper T cells) featured in 107 and 89 studies, respectively (Fig. 2C, D).

**Figure 2.**
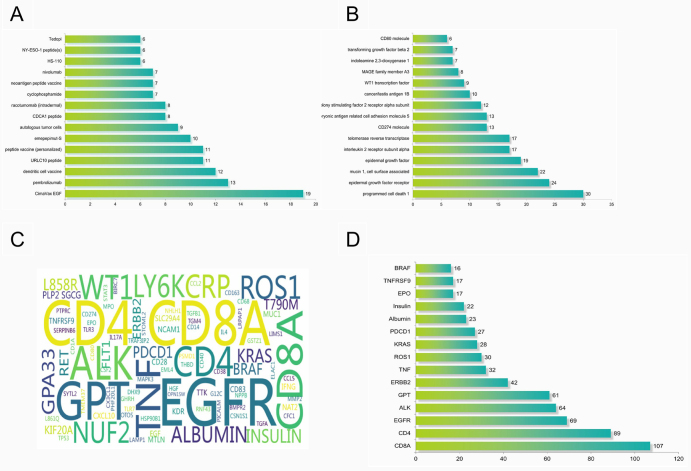
Mechanistic analysis of clinical trials for lung cancer vaccines. (A) Distribution of the top 15 most common drugs in clinical trials for lung cancer tumor vaccines. (B) Distribution of the top 15 most common targets in clinical trials for lung cancer vaccines. (C) Word cloud of biomarkers. (D) Distribution of the top 15 most common biomarkers in clinical trials for lung cancer vaccines.

In conclusion, overcoming current barriers to lung cancer vaccine development will require a deeply integrated ecosystem of interdisciplinary innovation, policy reform, and collaborative research. Only through synergistic efforts across academia, industry, and government can these promising therapies achieve full clinical translation and global impact.

## Data Availability

The datasets generated and analyzed during the current study are available in the INFORMA database (https://pharma.id.informa.com/).
